# QTL mapping and KASP marker development for seed vigor related traits in common wheat

**DOI:** 10.3389/fpls.2022.994973

**Published:** 2022-09-30

**Authors:** Zhankui Zeng, Cheng Guo, Xuefang Yan, Junqiao Song, Chunping Wang, Xiaoting Xu, Yuanfeng Hao

**Affiliations:** ^1^ College of Agronomy, Henan University of Science and Technology, Luoyang, China; ^2^ The Shennong Laboratory, Zhengzhou, China; ^3^ Institute of Crop Sciences, Chinese Academy of Agricultural Sciences (CAAS), Beijing, China

**Keywords:** KASP marker, QTL cluster, QTLs, seed vigor, *Triticum aestivum*

## Abstract

Seed vigor is an important parameter of seed quality, and identification of seed vigor related genes can provide an important basis for highly efficient molecular breeding in wheat. In the present study, a doubled haploid (DH) population with 174 lines derived from a cross between Yangmai16 and Zhongmai 895 was used to evaluate 10 seed vigor related traits in Luoyang during the 2018-2019 cropping season and in Mengjin and Luoning Counties during 2019-2020 cropping season for three environments. Quantitative trait locus (QTL) mapping of 10 seed vigor related traits in the DH population resulted in the discovery/identification of 28 QTLs on chromosomes 2B, 3D, 4B, 4D, 5A, 5B, 6A, 6B, 6D, 7A and 7D, explaining 3.6-23.7% of the phenotypic variances. Among them, one QTL cluster for shoot length, root length and vigor index was mapped between *AX-89421921* and *Rht-D1_SNP* on chromosome 4D in the physical intervals of 18.78-19.29 Mb (0.51 Mb), explaining 9.2-20.5% of the phenotypic variances. Another QTL for these traits was identified at the physical position 185.74 Mb on chromosome 5B, which was flanked by *AX-111465230* and *AX-109519938* and accounted for 8.0-13.3% of the phenotypic variances. Two QTLs for shoot length, shoot fresh weight and shoot dry weight were identified in the marker intervals of *AX-109384026-AX-111120402* and *AX-111651800-AX-94443918* on chromosomes 6A and 6B, explaining 8.2-11.7% and 3.6-10.3% of the phenotypic variance, respectively; both alleles for increasing phenotypic values were derived from Yangmai 16. We also developed the KASP markers for the QTL cluster *QVI.haust-4D.1/QSL.haust-4D/QRL.haust-4D*, and validated in an international panel of 135 wheat accessions. The germplasm, genes and KASP markers were developed for breeders to improve wheat varieties with seed vigor related traits.

## 1 Introduction

Seed vigor is a key parameter used to measure seed quality in wheat (Hao et al., 2006; Chen et al., 2016), and high-vigor seeds possess excellent characteristics such as fast growth, a high germination rate and strong stress resistance (Sun et al., 2007; Ventura et al., 2012). Therefore, seed vigor can significantly contribute to increasing grain yield and reducing agricultural production costs.

Numerous studies have attempted to detect seed vigor, including the standard germination test, the accelerated aging test, and the conductometry test (Xiang et al., 2020). Additionally, evaluation of volatile organic compounds is also a method for rapid identification of seed vigor (Umarani et al., 2020). Moreover, the research characters of seed vigor mainly include physiological indicators and morphological indicators, morphological indicators including vigor index, germination rate, germination energy, germination index, shoot length, root length, fresh and dry weight of the shoots and roots (Shi et al., 2020), and physiological indicators, including superoxide dismutase, catalase, peroxidase, and amylase (Han et al., 2018). Seed vigor related traits are complex quantitative traits controlled by multiple genes and are influenced by both genetic and environmental factors (Ma et al., 2004), such as soil fertility, maturity at harvest, and storage duration (He et al., 2016a; Liu et al., 2021).

With the rapid development of molecular biotechnology and sequencing technology, some QTLs for seed vigor have been identified in crops, and most of these studies have focused on rice (Hang et al., 2015; Zhang et al., 2017; Zhao et al., 2021a) and maize (Liu et al., 2018; Liu et al., 2020a; Wu et al., 2020). Eight QTLs for seed vigor have been identified by using a RIL population derived from a cross of ZS97 and MH63, among these QTLs, five QTLs (*qSV-1*, *qSV-5b*, *qSV-6a*, *qSV-6b*, and *qSV-11*) influenced seedling establishment, and three QTLs (*qSV-5a*, *qSV-5c* and *qSV-8*) influenced only germination (Xie et al., 2014). A total of 43 QTLs have been detected to control the early germination and seedling vigor traits in rice using the 167 BC_1_F_5_ selective introgression lines (Dimaano et al., 2020). Twenty-seven QTLs for germination and early seedling growth traits have been identified using 230 introgression lines in rice (Najeeb et al., 2020). Thirteen QTLs were found to control maize seed vigor on five chromosome regions under artificial aging conditions, using one inbred lines derived from a cross between X178 and I178 (Liu et al., 2018). A total of 18 QTLs for seed vigor were detected on four sweet corn germinations traits under artificial aging conditions, and a stable QTL was identified on chromosome 10 within an interval of 1.37 Mb, explaining 5.53-13.14% of the phenotypic variances (Wu et al., 2020). In wheat, QTL mapping studies of seed vigor related traits were limited, only a few studies have reported the QTLs associated with seed vigor. Thirty-seven QTLs for seedling related traits were located on 14 chromosomes, among which the QTLs on chromosomes 1D, 3B and 5D explained more than 20% of the phenotypic variances (Li et al., 2014b). In addition, about 20 QTLs for seed vigor, germination and early seedling growth were identified on chromosomes 1D, 2D, 4D, 5D and 7D under non-stress and osmotic conditions, explaining 11.6-37.3% of the phenotypic variances (Landjeva et al., 2010).

Although many QTLs for seed vigor related traits have been identified in crops, especially in rice and maize, relatively few genes associated with seed vigor have been successfully cloned. In rice, *OsLOX1* plays an important signaling role in the germination process by regulating the jasmonate pathway (Wang et al., 2008). *OsSAUR33* enhances seed vigor by regulating glucose metabolism during seed germination (Zhao et al., 2021b). Additionally, α-amylase gene expression is positively regulated by gibberellin in endosperm, and involved in seed vigor during seed germination (Damaris et al., 2019). In maize, *LOX-1* and *LOX-2* have the functions of decreasing seed vigor (Wu et al., 2020). *Tamyb10-1* not only controls grain color in wheat, but also influences the germination rate and germination index (Li et al., 2014a), and further, there are studies indicating that both *TaMyb10-A1* and *TaMyb10-D1* were associated with pre-harvest sprouting (PHS) resistance (Zhou et al., 2017; Yang et al., 2019), and *Myb10-D* confers PHS resistance by regulating ABA biosynthesis to delay germination in wheat (Lang et al., 2021).

Currently, seed vigor has not been directly selected as an important breeding trait in traditional wheat breeding programs, thus, the research progress has been slow. In this study, a doubled haploid (DH) population from Yangmai 16/Zhongmai 895 was developed using the maize-wheat hybridization. The DH lines and parents were planted at the Farm of Henan University of Science and Technology in Luoyang during 2018-2019 cropping season, and in Mengjin and Luoning Counties during 2019-2020 cropping season, and genotyped with the wheat 660K SNP array for QTL mapping of seed vigor-related traits. The aims were to 1) identify QTLs for seed vigor related traits, 2) explore the QTLs cluster, 3) preliminarily identify candidate genes based on multiple sequence alignments and gene annotation, and 4) develop KASP markers of the major loci for wheat breeding.

## 2 Materials and methods

### 2.1 Plant materials

A population of 174 DH lines derived from the cross of Yangmai 16/Zhongmai 895 and parents were planted at the Farm of Henan University of Science and Technology in Luoyang in 2018-2019 (2018XN, 34°62’N, 112°45’E) cropping season, and in Mengjin (2019MJ, 34°49’N, 112°26’E) and Luoning (2019LN, 34°38’N, 111°65’E) Counties in 2019-2020 cropping season.

An international panel of 135 wheat accessions was utilized for validation of the KASP markers, including 56 domestic wheat accessions and 79 cultivars from CIMMYT, USA, Australia, Canada and other countries representing global genetic diversities. The natural population was planted in Mengjin County and the Farm of Henan University of Science and Technology in 2020-2021 cropping season.

### 2.2 Field experimental design

The experiments were conducted in randomized block design with three replications. The plot size was 6.0 m^2^, and each line was sown in two rows, 20 cm apart and 3.0 m in length. Field management followed local standard of wheat cropping practices and cultivation conditions. The lines were harvested individually at the maturity stage when the moisture content was less than 13%. After harvest, the seeds were sun-dried under natural conditions to about 11% moisture content for phenotypic analysis. The data were collected within one month after crop harvesting.

### 2.3 Phenotypic determination

Seeds were germinated using the paper roll method (Huang et al., 2004), and the modified procedures are described as follows.

1. 25 healthy wheat seeds were selected randomly, and then were sterilized with 10% sodium hypochlorite solution for 5 min, the sterilized seeds were followed by rinsing with distilled water.
2. Germination paper was dampened with water at room temperature for 24 h, then two germination papers were placed completely together on the operating table.
3. 25 sterilized seeds were placed on the above germination papers at 3 cm from the upper edges of the germination papers with the embryo of seeds away from the upper edge of the germination papers to ensure the roots grew downwards. Moreover, the seeds were enenly distributed along the horizontal direction with 1.5 cm apart.
4. The germination papers with sterilized seeds were completely covered by another germination paper, and rolled them into a cylindrical form (starting at left side of germination papers and ending on the right side).
5. The germination papers with sterilized seeds, which have been rolled into a cylindrical form, were placed polyethylene bags, and then placed in an artificial climatic chamber with the seeds side up.

The temperature of the artificial climatic chamber was set to be 25°C with 16 h light/8 h dark. Ten traits were measured, including shoot length (SL), root length (RL), fresh shoot weight (FSW), fresh root weight (FRW), dry shoot weight (DSW), dry root weight (DRW), germination rate (GR), germination energy (GE), germination index (GI), and vigor index (VI). Among these traits, SL, RL, FSW and FRW were measured on 8^th^ day, then roots and shoots were dried in an oven at 80 °C to a constant weight and then measured the DSW and DRW. SL indicated the distance from sprout tip to seed embryo, and RL indicated the distance from seed embryo to root apical.

GR (%) = (Number of germinated seeds in 8 days/total number of seeds) × 100% ;

GE (%) = (Number of germinated seeds in 4 days/total number of seeds) × 100% ;

GI = ∑Gt/Dt ;

VI = GI×S ;

In the equation, Gt is the number of germinated seeds per day, Dt is the number of germination days, and S is the mean length of SL in each line.

### 2.4 Statistical analysis of the phenotyping data

Excel 2016 (Microsoft Corp., Redmond, USA) and SPSS (Version 22.0, IBM Corp., Armonk, NY, USA) were used for various statistical measures. Graphs were drawn using Origin 2022b (Originlab, Northampton, USA).

### 2.5 Genetic linkage map construction and QTL mapping

The DH lines and two parents were genotyped with an Affymetrix wheat 660K SNP array, and the genetic linkage map was constructed in an earlier report (Xu, 2019; Xu et al., 2020). The QTLs for seed vigor-related traits in three environments and the mean values were identified by inclusive composite interval mapping (ICIM) using the software QTL IciMapping v4.0. The walking step was set to 1 cM, the likelihood of odd (LOD) was set at 2.5, and the *P* value cutoff was 0.001 (Wang, 2009). A QTL, which was detected in two or more environments, was considered as a stable locus (Fu 2020). The QTLs were named following according to Rehman Arif and Börner, 2019 and McCouch et al., 1997.

The nucleotide sequences of the markers that were tightly linked with the QTLs were used for a BLAST search against the Chinese Spring genome from the International Wheat Genome Sequencing Consortium (IWGSC) to obtain their physical positions. Candidate genes within the QTL confidence intervals were predicted based on the reference genome and functional annotation information (Zeng et al., 2020; Wang et al., 2021).

### 2.6 KASP primer design and genotyping

Based on the mapping results, the sequences flanking the *QTL QVI.haust-4D.1/QSL.haust-4D/QRL.haust-4D* was used for designing KASP primers (PolyMarker, http://polymarker.tgac.ac.uk/). The primers were synthesized by Sangon Biotech (Shanghai) Co., Ltd. (Shanghai, China).

KASP reactions were run in a 4 μl volume, which contained 2 μl diluted DNA (30 ng/μl), 2 μl KASP master mix, and 0.045 μl primer mix (100 μM/μl). A total of 164 wheat lines were genotyped on an CFX 384 Real-Time System (BIO-RAD). The fluorescence signals of each reaction were collected, and was analyzed by BioRad CFX Manager Software.

## 3 Results

### 3.1 Phenotypic variation

**Figure 1:**
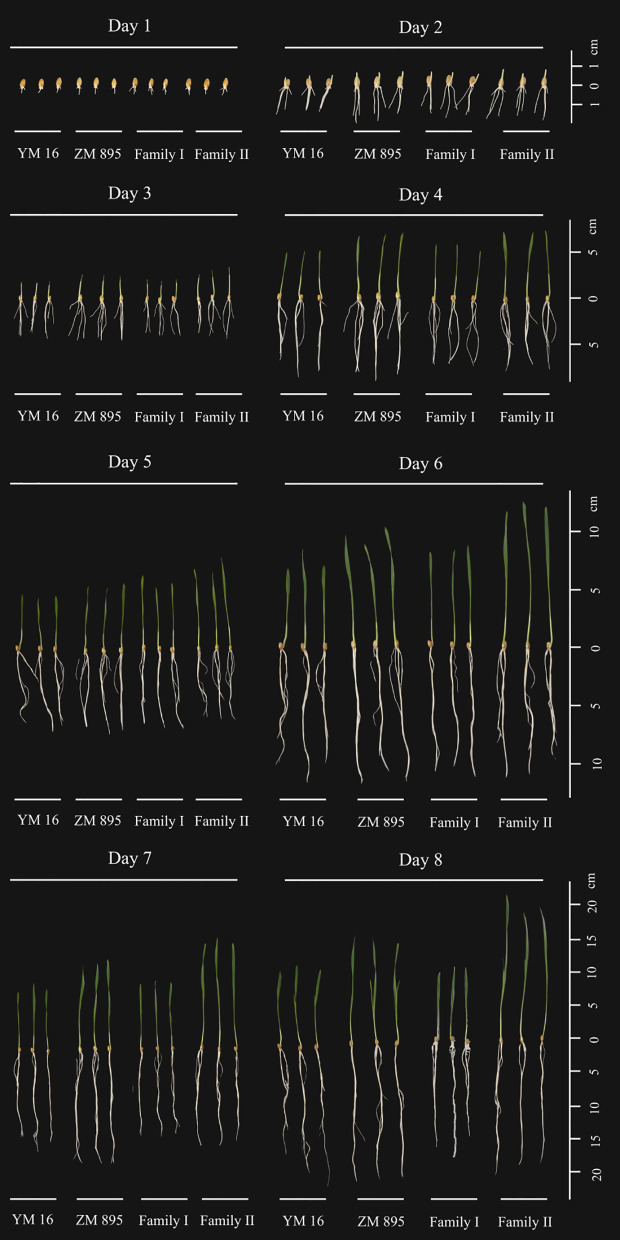
The growth of DH lines using the paper roll method.Three seeds are shown in each group, YM 16: Yangmai 16, ZM895: Zhongmai 895, Family I represent the lines of low seed vigor, Family II represent the lines of high seed vigor, Day1-Day 8 refer to days after germination.

**Figure 2:**
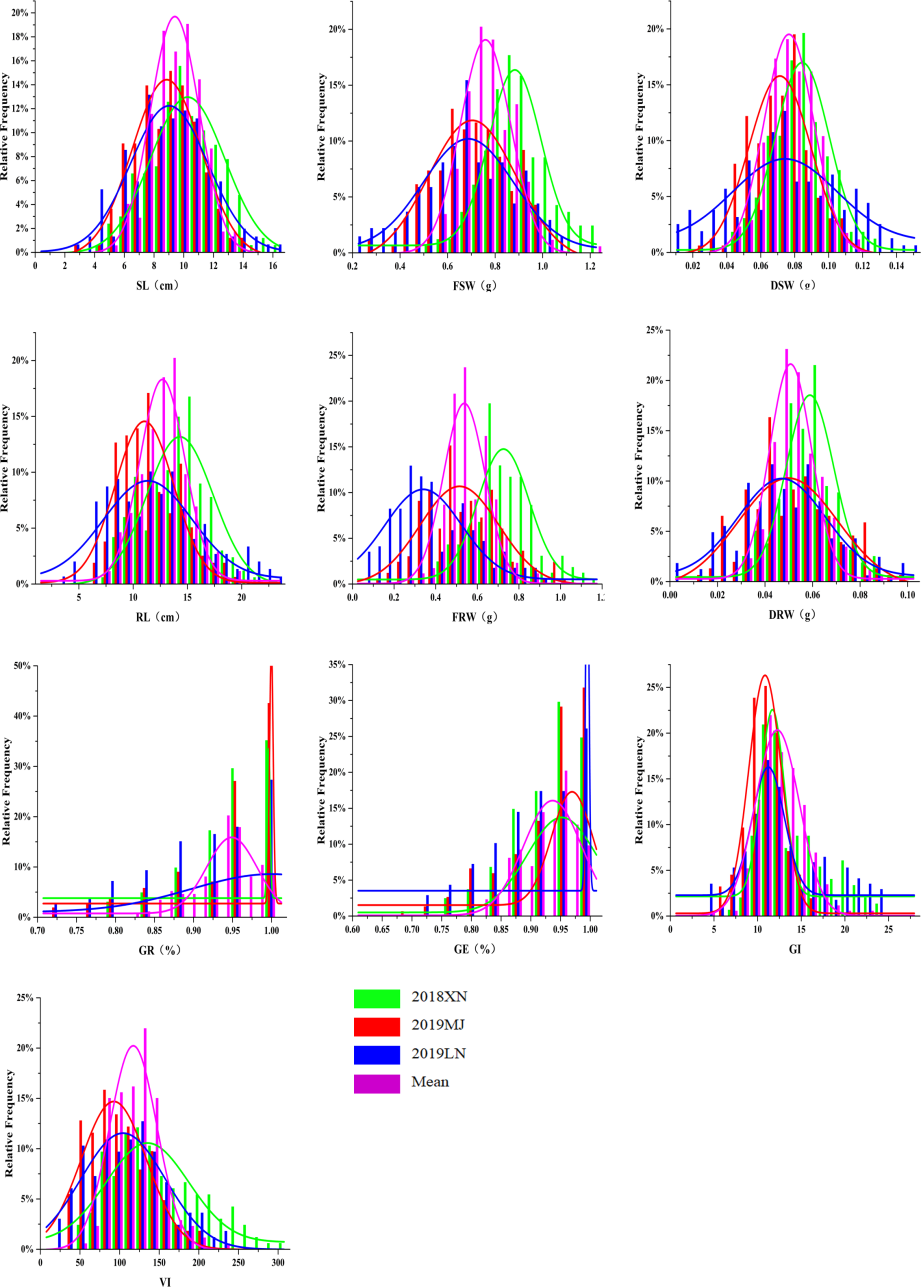
Frequency distribution for seed vigor related traits in DH population in three environments and mean values. Green, red, blue and purple represent 2018XN, 2019MJ, 2019LN and mean value, respectively. 2018XN, 2019MJ, 2019LN and mean represent data from the Farm of Henan University of Science and Technology in 2018-2019, Mengjin County in 2019-2020, Luoning County in 2019-2020, and mean values of three environments, respectively. SL, Shoot Length; RL, Root Length; FSW, Fresh Shoot Weight; FRW, Fresh Root Weight; DSW, Dry Shoot Weight; DRW, Dry Root Weight; GR, Germination Rate; GE, Germination Energy; GI, Germination Index; VI, Vigor Index.

The phenotypic data, including 10 traits of the DH lines and parents, are summarized in **Table 1**. Zhongmai 895 had higher levels of GR, GE, GI and VI than Yangmai 16 in 2018XN, 2019MJ, 2019LN and the mean values (**Figure 1**, **Table 1**). For FSW, FRW, DSW and DRW, the phenotypic values of the two parents were lower than the minimum values of the DH lines in all four environments. In addition, transgressive segregation was showed in SL, RL, GR, GE, GI and VI. The frequency distributions of SL, RL, FSW, FRW, DSW, DRW, GI, and VI among F5 DH lines showed a normal distribution (**Figure 2**) in all four environments from 2018 to 2020, indicating a typical quantitative character with multiple genes. Correlation analysis is shown in **Table 2**.

**Table 1:** Phenotypes of seed vigor related traits of Yangmai 16, Zhongmai 895, DH lines in three environments and the mean values.

Trait	Environment	Yangmai 16	Zhongmai 895	DH population		Trait	Environment	Yangmai 16	Zhongmai 895	DH population	
		$\bar{\text{x}}$	$\bar{\text{x}}$	$\bar{\text{x}}\pm s_{\bar{x}}$	Range			$\bar{\text{x}}$	$\bar{\text{x}}$	$\bar{\text{x}}\pm s_{\bar{x}}$	Range
SL (cm)	2018XN	10.24	10.31	10.21 ± 0.19	4.55-16.60	DRW (g)	2018XN	0.01	0.01	0.01 ± 0.001	0.03-0.12
	2019MJ	13.76	11.31	8.83 ± 0.16	3.13-14.06		2019MJ	0.01	0.01	0.01 ± 0.001	0.02-0.10
	2019LN	9.89	10.67	8.57 ± 0.21	1.68-16.57		2019LN	0.01	0.01	0.01 ± 0.002	0.02-0.10
	Mean	11.3	10.76	9.23 ± 0.41	5.85-13.45		Mean	0.01	0.01	0.05 ± 0.485	0.02-0.09
RL (cm)	2018XN	9.99	10.98	14.02 ± 0.23	7.69-21.36	GR (%)	2018XN	84	100	94.13 ± 0.01	56.00-100.00
	2019MJ	20.53	17.98	11.86 ± 0.23	6.25-22.03		2019MJ	60	92	92.34 ± 0.01	44.00-100.00
	2019LN	15.28	15.94	11.50 ± 0.35	3.78-23.41		2019LN	72	96	84.80 ± 0.01	20.00-100.00
	Mean	15.27	14.97	12.28 ± 0.43	6.57-19.81		Mean	72	96	93.63 ± 0.23	72.00-100.00
FSW (g)	2018XN	0.08	0.08	0.09 ± 0.011	0.42-1.29	GE (%)	2018XN	84	96	92.02 ± 0.01	52.00-100.00
	2019MJ	0.1	0.08	0.07 ± 0.012	0.23-1.01		2019MJ	60	92	90.3 ± 0.01	32.00-100.00
	2019LN	0.09	0.08	0.06 ± 0.018	0.12-1.09		2019LN	72	96	84.38 ± 0.01	20.00-100.00
	Mean	0.09	0.08	0.71 ± 0.41	0.40-0.96		Mean	72	94.67	92.78 ± 0.23	80.00-100.00
FRW (g)	2018XN	0.04	0.04	0.08 ± 0.013	0.44-1.32	GI	2018XN	9.75	11.25	14.78 ± 0.31	7.76-24.50
	2019MJ	0.1	0.09	0.05 ± 0.014	0.15-1.02		2019MJ	6.5	15	11.00 ± 0.20	3.96-19.83
	2019LN	0.05	0.06	0.04 ± 0.015	0.16-0.93		2019LN	13.33	18.67	13.29 ± 0.37	4.25-24.33
	Mean	0.06	0.06	0.53 ± 0.475	0.30-0.85		Mean	9.86	14.97	12.10 ± 0.43	6.86-24.00
DSW (g)	2018XN	0.01	0.01	0.01 ± 0.001	0.03-0.15	VI	2018XN	99.84	115.99	152.84 ± 4.72	52.83-388.57
	2019MJ	0.01	0.01	0.01 ± 0.001	0.02-0.11		2019MJ	73.52	206.4	99.73 ± 3.14	30.57-231.99
	2019LN	0.01	0.01	0.01 ± 0.002	0.01-0.17		2019LN	131.87	199.17	114.08 ± 4.21	23.74-298.26
	Mean	0.01	0.01	0.08 ± 0.452	0.03-0.11		Mean	101.74	173.85	114.80 ± 0.52	34.36-238.56

2018XN, 2019MJ, 2019LN and Mean represent data from the Farm of Henan University of Science and Technology in 2018-2019, Mengjin County in 2019-2020, Luoning County in 2019-2020, and mean values of three environments, respectively. SL, Shoot Length; RL, Root Length; FSW, Fresh Shoot Weight; FRW, Fresh Root Weight; DSW, Dry Shoot Weight; DRW, Dry Root Weight; GR, Germination Rate; GE, Germination Energy; GI, Germination Index; VI, Vigor Index.

**Table 2:** Correlations among seed vigor related traits in the Yangmai 16/Zhongmai 895 DH population.

	SL	RL	FSW	FRW	DSW	DRW	GR	GE	GI
RL	0.741^**^								
FSW	0.741^**^	0.624^**^							
FRW	0.557^**^	0.617^**^	0.682^**^						
DSW	0.690^**^	0.606^**^	0.911^**^	0.651^**^					
DRW	0.684^**^	0.674^**^	0.708^**^	0.809^**^	0.695^**^				
GR	0.043	-0.021	-0.041	-0.033	-0.105	-0.032			
GE	0.040	0.021	-0.081	-0.001	-0.082	-0.042	0.758^**^		
GI	0.210^**^	0.186^*^	0.144	0.141	0.114	0.132	0.405^**^	0.435^**^	
VI	0.692^**^	0.532^**^	0.505^**^	0.041^**^	0.424^**^	0.505^**^	0.306^**^	0.281^**^	0.724^**^

* and **, significant at *P*<0.05 and *P*<0.01, respectively.

SL, Shoot Length; RL, Root Length; FSW, Fresh Shoot Weight; FRW, Fresh Root Weight; DSW, Dry Shoot Weight; DRW, Dry Root Weight; GR, Germination Rate; GE, Germination Energy; GI, Germination Index; VI, Vigor Index.

### 3.2 QTL analysis of seed vigor related traits

#### 3.2.1 Shoot length and root length

**Figure 3:**
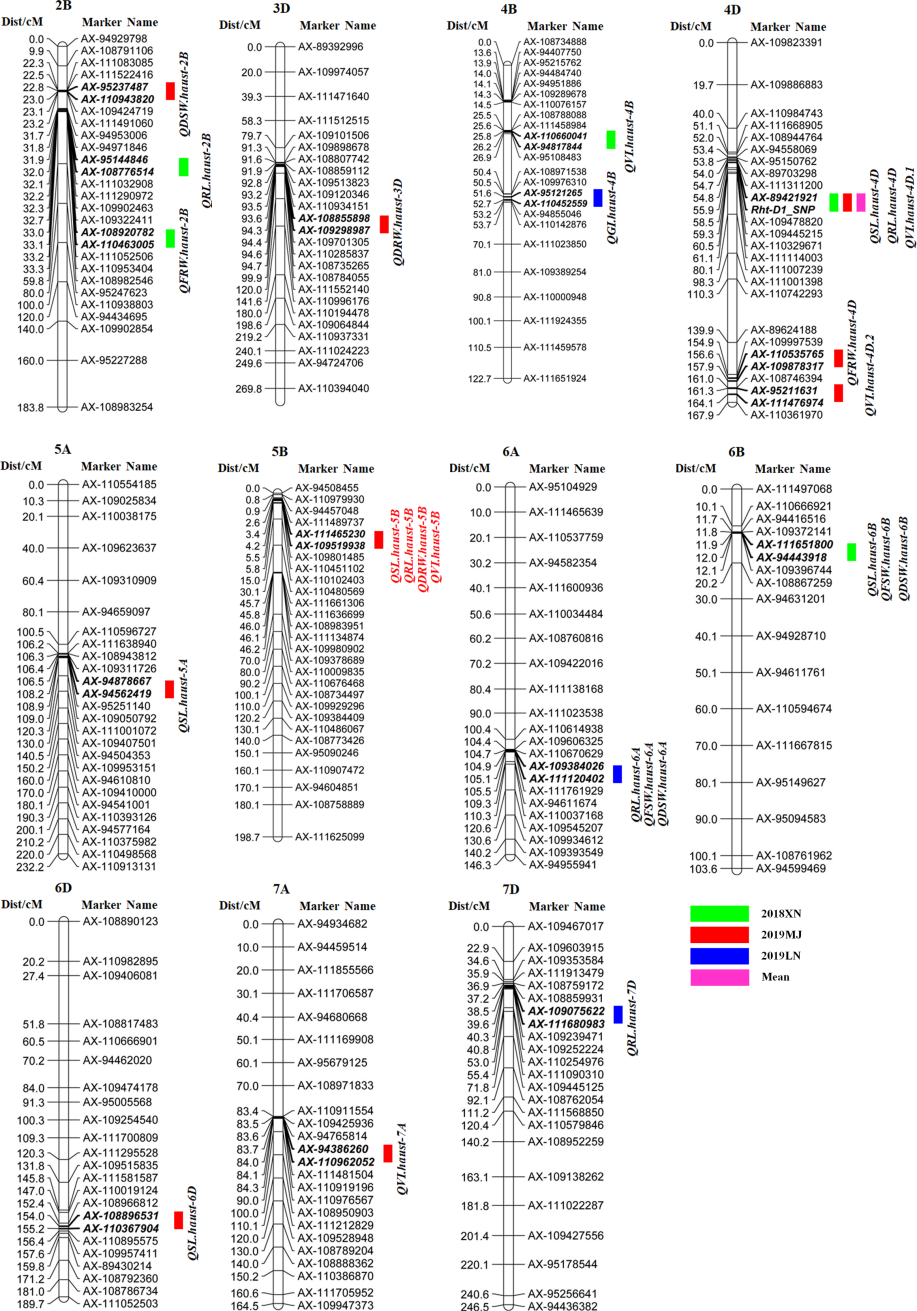
QTL mapping for seed vigor related traits in the Yangmai 16/Zhongmai 895 DH population. Markers’ names are shown on the right of vertical axis, and their genetic positions are shown in cM on the left. Green, red, blue and purple represent QTL mapped using data of 2018XN, 2019MJ, 2019LN and mean values, respectively.

Twenty-five QTLs were detected for seed vigor-related traits on chromosomes 2B, 3D, 4B, 4D, 5A, 5B, 6A, 6B, 6D, 7A, and 7D, explaining 3.6-23.7% of the phenotypic variances (**Table 3**, **Figure 3**). Among those QTLs, five QTLs for SL were detected on chromosomes 4D, 5A, 5B, 6B and 6D, respectively. *QSL.haust-4D* in the marker intervals *AX-89421921-Rht-D1_SNP* explained 21.1-23.7% of the phenotypic variances, and was detected in 2019MJ and the mean value environment, the alleles for increasing SL came from Zhongmai 895. *QSL.haust-5A* was detected in a single environment and it was in the marker intervals *AX-94878667-AX-94562419*, explaining 9.6% of the phenotypic variance. *QSL.haust-5B* was located in the marker intervals *AX-111465230-AX-109519938*, explaining 10.3% of the phenotypic variances. *QSL.haust-6B* and *QSL.haust-6D* were detected in a single environment and in the marker intervals *AX-111651800-AX-94443918* and *AX-108896531-AX-110367904*, explaining 3.6-5.2% of the phenotypic variance, respectively. The alleles of *QSL.haust-5A*, *QSL.haust-5B*, *QSL.haust-6B* and *QSL.haust-6D* for increasing SL were all derived from Yangmai 16.

**Table 3:** QTL mapping for seed vigor related traits in the Yangmai 16/Zhongmai 895 DH population.

Trait	QTL	Environment	Chromosome	Physical position/Mb	Marker interval	LOD	PVE (%)	Add^#^
SL	*QSL.haust-4B*	2018XN	4B	–	*AX-94484740-AX-94951886*	21.8	29.4	-1.32
	*QSL.haust-4D.1*	2018XN	4D	15.91	*AX-108944764-AX-94558069*	30	45.1	1.62
	*QSL.haust-4D.2*	2019MJ	4D	18.78-19.29	*AX-89421921-Rht-D1_SNP*	11.8	23.7	1.02
		Mean	4D	18.78-19.29	*AX-89421921-Rht-D1_SNP*	9.3	21.1	0.69
	*QSL.haust-5A*	2019LN	5A	502.80-658.87	*AX-94878667-AX-94562419*	4	9.6	-0.88
	*QSL.haust-5B.1*	2019MJ	5B	185.74	*AX-111465230-AX-109519938*	5.4	10.3	-0.68
	*QSL.haust-5B.2*	2018XN	5B	300.01-305.34	*AX-108983951-AX-111134874*	31.7	49	1.68
	*QSL.haust-6B*	2018XN	6B	18.85	*AX-111651800-AX-94443918*	3.6	3.6	-0.45
	*QSL.haust-6D*	2019MJ	6D	450.70-451.08	*AX-108896531-AX-110367904*	3	5.2	-0.48
RL	*QRL.haust-2B*	2018XN	2B	30.47	*AX-95144846-AX-108776514*	4.8	8.9	-0.88
	*QRL.haust-4D*	2018XN	4D	18.78-19.29	*AX-89421921-Rht-D1_SNP*	9.9	20.6	1.35
		Mean	4D	18.78-19.29	*AX-89421921-Rht-D1_SNP*	4.4	10.7	0.7
	*QRL.haust-5B*	2019MJ	5B	185.74	*AX-111465230-AX-109519938*	3.9	10.5	-0.92
	*QRL.haust-6A*	2019LN	6A	599.03-600.46	*AX-109384026-AX-111120402*	4.9	11.7	-1.55
	*QRL.haust-7D*	2019LN	7D	18.87-20.33	*AX-109075622-AX-111680983*	3.2	7.7	-1.26
FSW	*QFSW.haust-6A*	2019LN	6A	599.03-600.46	*AX-109384026-AX-111120402*	3.4	8.2	-0.08
	*QFSW.haust-6B*	2018XN	6B	18.85	*AX-111651800-AX-94443918*	3.9	10.3	-0.04
FRW	*QFRW.haust-2B*	2018XN	2B	35.90-37.43	*AX-108920782-AX-110463005*	3.8	10.3	-0.05
	*QFRW.haust-4D*	2019MJ	4D	–	*AX-110535765-AX-109878317*	3.4	8.5	-0.05
DSW	*QDSW.haust-2B*	2019MJ	2B	5.32	*AX-95237487-AX-110943820*	3.1	7.9	0.01
	*QDSW.haust-6A*	2019LN	6A	599.03-600.46	*AX-109384026-AX-111120402*	3.9	9.3	-0.01
	*QDSW.haust-6B*	2018XN	6B	18.85	*AX-111651800-AX-94443918*	3.1	7.9	-0.0047
DRW	*QDRW.haust-3D*	2019MJ	3D	167.55	*AX-108855898-AX-109298987*	3.2	7.3	0.0048
	*QDRW.haust-5B*	2019MJ	5B	185.74	*AX-111465230-AX-109519938*	5.3	13.3	-0.01
GI	*QGI.haust-4B*	2019LN	4B	289.61-453.26	*AX-95121265-AX-110452559*	3.3	9.5	-1.5
VI	*QVI.haust-4B*	2018XN	4B	27.52	*AX-110660041-AX-94817844*	6	14.3	-21.47
	*QVI.haust-4D.1*	2018XN	4D	18.78-19.29	*AX-89421921-Rht-D1_SNP*	5.5	12.9	20.47
		2019MJ	4D	18.78-19.29	*AX-89421921-Rht-D1_SNP*	5.5	8.7	11.62
		Mean	4D	18.78-19.29	*AX-89421921-Rht-D1_SNP*	4.4	9.5	9.19
	*QVI.haust-4D.2*	2019MJ	4D	–	*AX-95211631-AX-111476974*	7.5	12	-13.77
	*QVI.haust-5B*	2019MJ	5B	185.74	*AX-111465230-AX-109519938*	4.8	8	-11.22
	*QVI.haust-7A*	2019MJ	7A	269.02-545.69	*AX-94386260-AX-110962052*	4.7	7.9	11.23

^#^The favorable alleles at loci with negative additive effects were derived from Yangmai 16 and those with positive additive effects were derived from Zhongmai 895, ‘-’ indicates that physical positions are not available.

2018XN, 2019MJ, 2019LN and mean represent data from the Farm of Henan University of Science and Technology in 2018-2019, Mengjin County in 2019-2020, Luoning County in 2019-2020, and mean values of three environments, respectively.

SL, Shoot Length; RL, Root Length; FSW, Fresh Shoot Weight; FRW, Fresh Root Weight; DSW, Dry Shoot Weight; DRW, Dry Root Weight; GR, Germination Rate; GE, Germination Energy; GI, Germination Index; VI, Vigor Index.

Five QTLs for RL were observed on chromosomes 2B, 4D, 5B, 6A, and 7D, respectively (**Table 3**, **Figure 3**), *QRL.haust-4D* was mapped on chromosome 4D in two environments and flanked by markers *AX-89421921* and *Rht-D1_SNP*, explaining 10.7% and 20.6% of the phenotypic variances, and the alleles for increasing RL were derived from Zhongmai 895. *QRL.haust-2B*, *QRL.haust-5B*, *QRL.haust-6A*, and *QRL.haust-7D* were only found in single environments in the marker intervals *AX-95144846-AX-108776514*, *AX-111465230-AX-109519938*, *AX-109384026-AX-111120402*, and *AX-109075622-AX-111680983*, respectively. The phenotypic variances explained by these QTLs were 8.9%, 10.5%, 11.7%, and 7.7%, respectively. The alleles for increasing RL were all originated from Yangmai 16.

#### 3.2.2 Shoot fresh weight and root fresh weight

Two QTLs (*QFSW.haust-6A* and *QFSW.haust-6B*) for FSW were detected on chromosomes 6A and 6B in two environments (**Table 3**, **Figure 3**), locating in the marker intervals of *AX-109384026-AX-111120402* and *AX-111651800-AX-94443918*, accounting for 8.2% and 10.3% of the phenotypic variances, respectively. The alleles for increasing FSW were derived from Yangmai 16.

Two QTLs (*QFRW.haust-2B* and *QFRW.haust-4D*) for FRW were mapped in the marker intervals of *AX-108920782-AX-110463005* and *AX-110535765-AX-109878317* on chromosomes 2B and 4D in single environments, explaining 10.3% and 8.5% of the phenotypic variance, respectively. The alleles for increasing FRW were originated from Yangmai 16.

#### 3.2.3 Shoot dry weight and root dry weight

Three QTLs for DSW were mapped on chromosomes 2B, 6A, and 6B (**Table 3**, **Figure 3**) in 2019MJ, 2019LN and 2018XN, respectively. *QDSW.haust-2B* with the allele for increasing DSW from Zhongmai 895 was closely linked with *AX-95237487* and *AX-110943820*, explaining 7.9% of the phenotypic variance. *QDSW.haust-6A* and *QDSW.haust-6B* in the marker intervals *AX-109384026-AX-111120402* and *AX-111651800-AX-94443918* accounted for 9.3% and 7.9% of the phenotypic variances, respectively. The alleles for increasing DSW came from Yangmai 16.

Two QTLs for DRW were detected on chromosomes 3D and 5B, respectively. *QDRW.haust-3D* flanked by the markers *AX-108855898* and *AX-109298987* explained 7.3% of the phenotypic variance, and the alleles for increasing DRW was derived from Zhongmai 895. *QDRW.haust-5B* was only mapped in 2019MJ. It was located in the marker interval *AX-111465230-AX-109519938*, accounting for 13.3% of the phenotypic variance, and the allele for increasing DRW was derived from Yangmai 16.

#### 3.2.4 Germination index and vigor index

Only one QTL for GI (*QGI.haust-4B*) was detected on chromosome 4B in 2019LN (**Table 3**, **Figure 3**), which was closely linked with markers *AX-95121265* and *AX-110452559*, explaining 9.5% of the phenotypic variance, and the allele for increasing GI was originated from Yangmai 16.

Five QTLs for VI were mapped on chromosomes 4B, 4D, 5B and 7A, respectively (**Table 3**, **Figure 3**). *QVI.haust-4D.1* was mapped between markers *AX-89421921* and *Rht-D1_SNP* in three environments, accounting for 8.7-12.9% of the phenotypic variances. The allele for increasing VI was contributed by Zhongmai 895. Three QTLs (*QVI.haust-4B*, *QVI.haust-4D.2* and *QVI.haust-5B*) were detected on chromosomes 4B, 4D and 5B in the marker intervals *AX-110660041-AX-94817844*, *AX-95211631-AX-111476974* and *AX-111465230-AX-109519938* explained 14.3%, 12.0% and 8.0% of the phenotypic variances, respectively. The alleles for increasing VI were derived from Yangmai 16. *QVI.haust-7A* closely linked with markers *AX-94386260* and *AX-110962052* accounted for 8.0% of the phenotypic variance. The allele for increasing VI came from Zhongmai 895.

### 3.3 Effect of QTL cluster for seed vigor related traits

**Figure 4:**
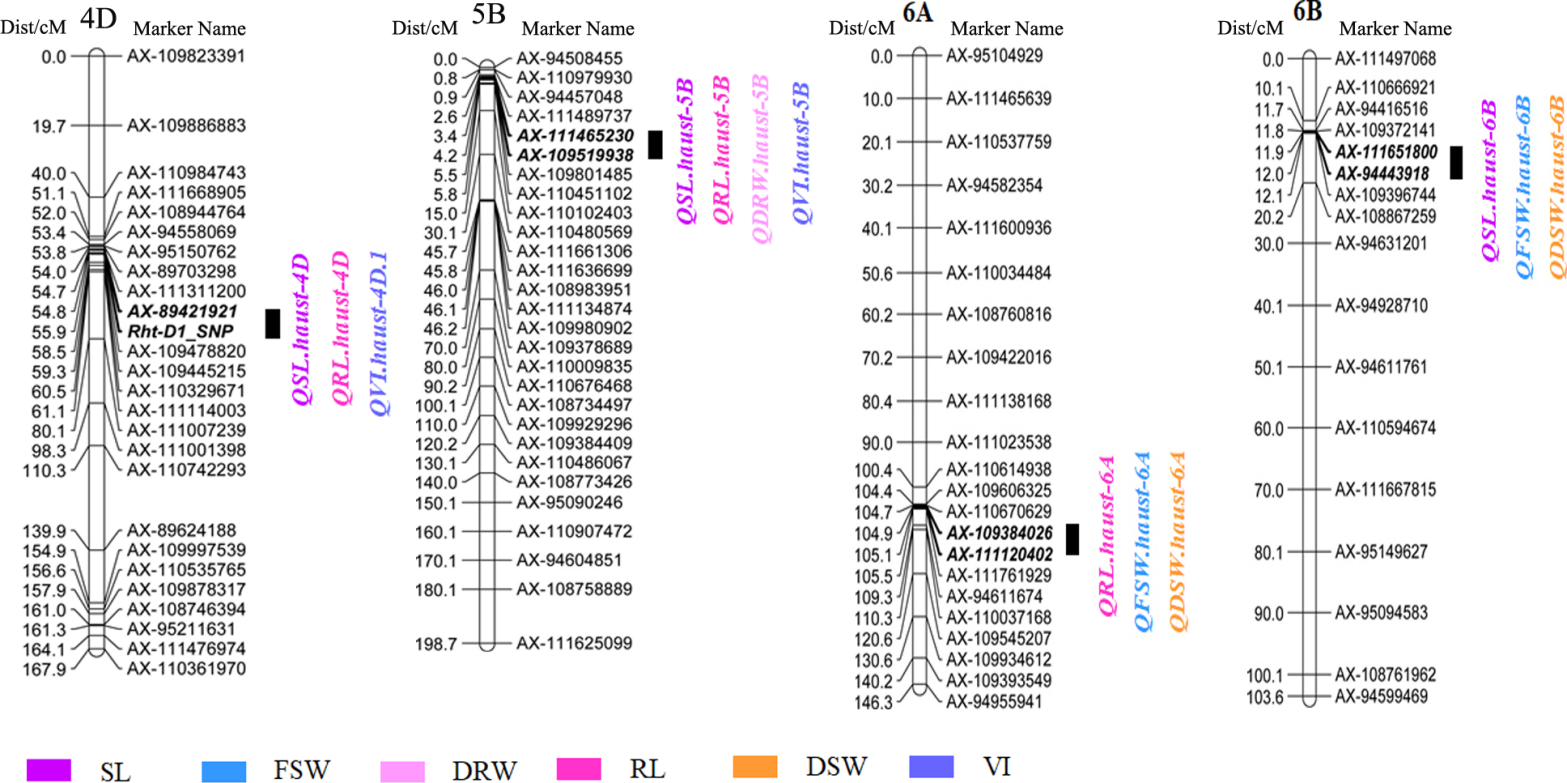
QTL clusters in the Yangmai 16/Zhongmai 895 DH population.

We detected four QTL clusters on chromosomes 4D, 5B, 6A and 6B (**Table 3**, **Figure 4**). The QTL flanked by markers *AX-89421921* and *Rht-D1_SNP* on chromosome 4D was associated with SL, RL, and VI, accounting for 8.7-23.7% of the phenotypic variances. The alleles of the QTL for increasing SL, RL, and VI were derived from Zhongmai 895. The QTL closely linked to markers *AX-111465230* and *AX-109519938* on chromosome 5B was associated with SL, RL, DRW and VI, explaining 8.0-13.3% of the phenotypic variances. The alleles for increasing SL, RL, DRW and VI were originated from Yangmai 16. Another QTL for RL, FSW and DSW on chromosome 6A was flanked by markers *AX-109384026* and *AX-111120402*, contributing 8.2-11.7% of the phenotypic variances. Its alleles for increasing RL, FSW and DSW came from Yangmai 16. The QTL tightly linked with markers *AX-111651800* and *AX-94443918* on chromosome 6B was associated with SL, FSW and DSW, accounting for 3.6-10.3% of the phenotypic variances. The alleles for increasing SL, FSW and DSW were derived from Yangmai 16.

### 3.4 KASP marker development of *QVI.haust-4D.1/QSL.haust-4D/QRL.haust-4D*

**Figure 5:**
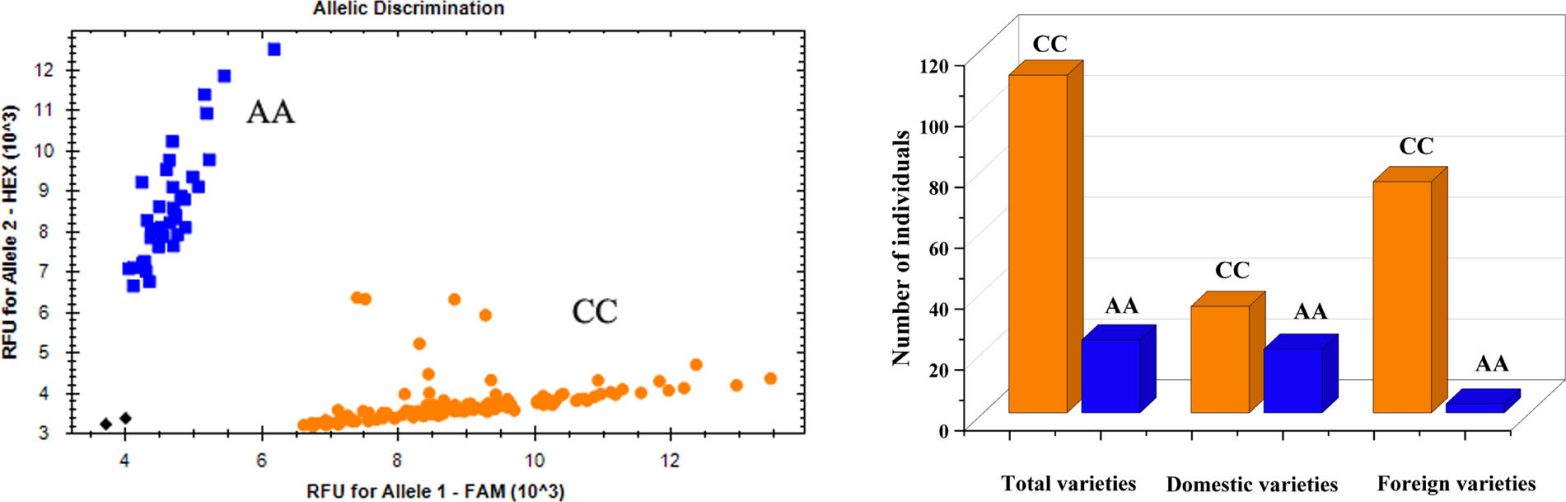
Genotype calling screenshots of the KASP marker. Orange indicates the C allele of Yangmai 16, blue indicates the A allele of Zhongmai 895, and black indicates the blank control. The same below.

For effective utilization of the major QTL in plant breeding, KASP markers closely linked to the QTL cluster *QVI.haust-4D.1/QSL.haust-4D/QRL.haust-4D* were developed (**Table 4**), and used to test the genotypes of the natural population (**Figure 5**).

**Table 4:** The primer sequences of KASP marker for *QVI.haust-4D.1/QSL.haust-4D/QRL.haust-4D*.

QTL	Primer	Allele	Primer sequence (5′-3′)
*QVI.haust-4D.1/* *QSL.haust-4D.2* */QRL.haust-4D*	*KASP-4D-A*	C/A	GAAGGTGACCAAGTTCATGCTCATGGCCATCTCGAGCTGCTC
	*KASP-4D-B*		GAAGGTCGGAGTCAACGGATTCATGGCCATCTCGAGCTGCTA
	*KASP-4D-C*		CGGGTACAAGGTGCGCGCC

The underlined parts represent the fluorescent junction sequence.

A and B in primer names indicate Yangmai 16 and Zhongmai 895 allele-specific primers, respectively, and C is the common reverse primer.

**Figure 6:**
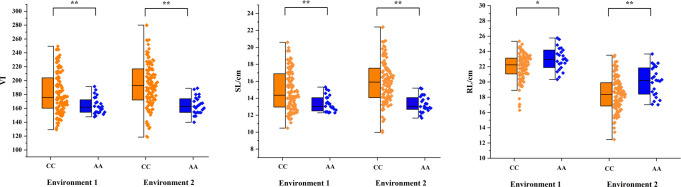
Allelic effects identified by the KASP marker in the natural population. Blue indicates the A allele of Zhongmai 895, and orange indicates the C allele of Yangmai 16. * and **, significant at *P<*0.05 and *P<*0.01, respectively.

Among the 135 wheat varieties, there were 111 CC (82.2%) and 24 AA (17.8%) genotypes (**Figure 5**), and VI, SL and RL were significantly different (*P*<0.01 or *P*<0.05) between CC group and AA group (**Figure 6**). Meanwhile, the CC genotype (Yangmai 16) had higher VI and SL than the AA genotype (Zhongmai 895), whereas the RL showed the opposite effect. Among the domestic varieties, there were 35 CC genotypes and 21 AA genotypes, respectively. There were 76 CC genotypes and 3 AA genotypes, respectively among 79 foreign wheat varieties.

## 4 Discussion

To improve sprouting ratio and seedling quality, numerous studies have mapped seed related QTLs and genes in crops such as seed longevity (2020; Rehman Arif and Börner, 2019; Rehman Arif et al., 2022), seed dormancy (2021; Rikiishi et al., 2010), pre-harvest sprouting (PHS) (Lang et al., 2021; Tai et al., 2021), these traits are closely related to seed vigor. Studies have revealed that seed aging and deterioration could induce the loss of seed vigor during storage (Mao et al., 2018), and seed dormancy is an important trait preventing PHS (Layat et al., 2021). Two PIMT genes are involved in both seed longevity and seed germination vigor in rice and Arabidopsis (Wei et al., 2015; Tang et al., 2020). These studies provided a rich and instructive backdrop for further research of seed vigor in wheat. However, we also should note that few studies have directly evaluated the QTL of wheat seed vigor, likely due to the diversity of evaluation indices and measurement methods of seed vigor, as well as the extremely large and complex genome of wheat.

### 4.1 QTL cluster on Chromosome 4D

**Figure 7:**
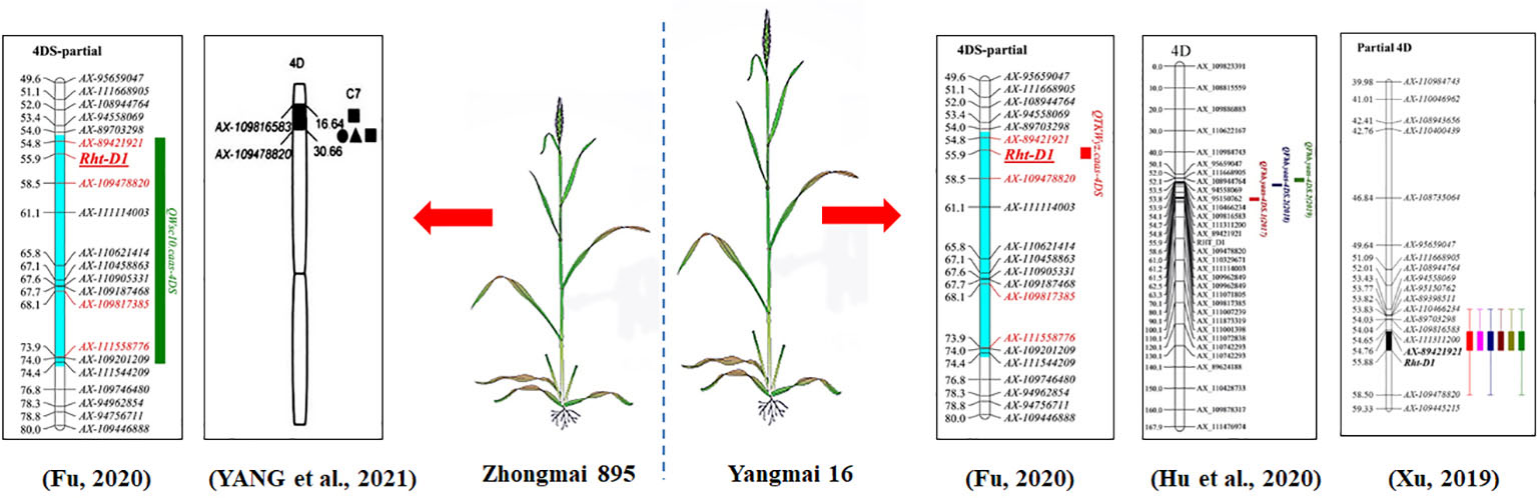
QTLs of the DH population on chromosome 4D.

A QTL hotspot region with SL, RL and VI was detected on chromosome 4D in different environments and was tightly linked with markers *AX-89421921* and *Rht-D1_SNP* in the physical interval of 18.78-19.29 Mb (0.51 Mb) (**Figure 7**), and the alleles of this QTL for increasing SL, RL and VI were derived from Zhongmai 895. Studies have revealed that the D genome has many trait loci for early seedling vigor related traits (Villar et al., 1998; Bultynck et al., 2004; Margreet et al., 2005), seed longevity (Rehman Arif et al., 2012; Agacka-Mołdoch et al., 2016) and PHS resistance (Himi et al., 2011; Lang et al., 2021). For instance, the common wheat introgression lines that carry the D genome fragmentation of Ae. tauschii have been used for QTL mapping for seed vigor related traits by Landjeva et al. (2010). Twenty QTLs were mapped on chromosomes 1D, 2D, 4D, 5D and 7D, the QTLs mapped on 1D and 5D showed that they were important sites for genes affecting seed longevity related traits, and the QTLs on chromosome 4D for early seedling vigor related traits were tightly linked with markers *Xgdm129* and *Xgdm61*. Quantitative trait loci for PHS resistance were detected on chromosome 3D in wheat (*PHS-3D/Myb10-D*), and it confered PHS resistance by regulating ABA biosynthesis to delay germination in wheat (Lang et al., 2021; Tai et al., 2021). A QTL-hotspot region has been mapped on the short arm of chromosome 4D (Wang, 2017). It was associated with SL, RL, and VI, and located near marker *Xgwm624* in physical position 34.7 Mb. However, the physical positions/intervals were different locus between the above QTLs and the QTL cluster on chromosome 4D in this study, and we speculated that the QTL cluster on chromosome 4D in this study was a new gene for seed vigor.

On the other hand, QTL mapping for other traits have been reported using this DH population. A stable QTL for thousand kernel weight (*QTKWyz.caas-4DS*) was identified on chromosome 4D. *QWsc10.caas-4DS* was also mapped on chromosome 4D and the alleles increasing thousand kernel weight (TKW) and water soluble carbohydrates (WSC) were derived from Zhongmai 895 (Fu et al., 2019; Fu et al., 2020). A Fusarium head blight (FHB) resistance QTL (*QFhb.yaas-4DS*) and a powdery mildew resistance QTL have also been reported on chromosome 4D in this population (Hu et al., 2019; Xu et al., 2020), both of which were contributed by Yangmai 16. Yang et al. (2021) also identified a QTL for root traits on chromosome 4D with the allele for increasing root traits from Zhongmai 895 (**Figure 7**). The QTL intervals might play an important role in maintaining seed vigor, plant height, yield-related traits, and disease resistance.

Furthermore, we searched the genes within the locus on 4D, and three high confidence genes were identified (IWGSC RefSeq v1.0), including *TraesCS4D02G040400*, *TraesCS4D02G040500* and *TraesCS4D02G040700*. *TraesCS4D02G040400* reduce the plant height of wheat (Wilhelm et al., 2013; Liu et al., 2020b), but it is also associated with the levels of resistance to FHB (Lu et al., 2011; Steiner et al., 2017; Jones et al., 2018), and reduced anther extrusion (Buerstmayr and Buerstmayr, 2016).

Further, the nonmutant types of *RHT-B1* or *RHT-D1* genes have longer root lengths (Mohan, et al., 2021). Certainly, other genes of RHT family were found to have similar functions as well. For example, studies have proven that the *Rht-B1b* and *Rht-D1b* have effects on FHB susceptibility and reduce anther extrusion (Holzapfel et al., 2008; He et al., 2016b; Prat et al., 2016; Jones et al., 2018). *Rht3* has a significant and negative affect on plant height, biomass, coleoptile length, and seedling establishment (Ellis et al., 2004; Addisu et al., 2008; Mohan et al., 2021). It is generally known that coleoptile length is governed by multiple genes and has a strong additive effect and high heritability (Rebetzke et al., 2007; Murphy et al., 2008). The *Rht1/Rht2* mutations not only affect stem elongation but also negatively affect coleoptile length (Botwright et al., 2001). A slight effect of Rht loci on root length and density has shown (Manske et al., 2002). However, there were also different views which were *Rht-B1b* and *Rht-D1b* negatively affect seedling root length (Nagel et al., 2013). Moreover, allelic variation of the *Rht-D1* locus had a significant correlation with coleoptile length (Khadka et al., 2021).

*RHT-D1* encodes a DELLA protein, which is a key negative regulator of gibberellin signaling, functioning as a transcriptional activator in wheat (Daviere and Achard, 2013), and gibberellin is a major hormone that promotes growth, thereby promoting seed germination and seedling growth (Feng, 2015; Zuo et al, 2021). Meanwhile, *Rht-D1a* is significantly associated with alpha-amylase levels, and amylase content is one of the important indexes of seed vigor (Liu et al, 2021). *AT1G14920* (GAI) is an orthologue of *RHT-D1* in Arabidopsis that mediate GA regulation of stem elongation, promoting the germination of Arabidopsis seeds. The Arabidopsis DELLAs have been demonstrated to have similar functions in wheat and display relatively discrete (e.g., *RGL2* in seed germination) gibberellic acid (GA) response regulation functions (Harberd et al., 2009); *RGL2* is closely related to GAI and regulates seed germination in response to GA (Lee et al., 2002). DELLA proteins repressed the GA responses and have been proposed to act by inhibiting the activity of transcription factors (Ogawa et al., 2003; de Lucas et al., 2008). In conclusion, we speculated that the locus on chromosome 4D was the *RHT-D1* for seed vigor related traits.

### 4.2 QTL cluster on Chromosome 5B

In this study, a QTL for SL, RL, DRW and VI was found to be tightly linked with markers *AX-111465230* and *AX-109519938*, and the allele of this QTL for increasing SL, RL, DRW and VI was derived from Yangmai 16. In previous studies, it has been reported that one QTL hotspot region for six seed vigor related traits was identified on the long arm of chromosome 5B in the DH population derived from the cross of Hanxuan 10 and Lumai 14; it was closely linked with *AX-94643729* and *AX-110529646* in a physical position near 710.96 Mb (Shi et al., 2020) and differs from the 5B QTL in this study. Two QTL hotspot regions with epistatic effects were also found on chromosome 5B, which were associated with SL, RL, and VI, and tightly flanked by *Xwmc616* and *Xwmc740* in a physical position near 70.0 Mb (Wang, 2017). The QTL is also different from the 5B QTL in this study based on the physical positions, indicating that the 5B QTL identified in this study is likely to be a new locus for seed vigor related traits.

### 4.3 QTL clusters on chromosomes 6A and 6B

Two QTL hotspot regions for SL, FSW, and DSW were detected in the physical intervals of 599.03-600.46 Mb and near the 18.85 Mb on chromosomes 6AL and 6BS, respectively. The QTL hotspot region for VI and GI was associated with the marker *Xwmc201* on the short arm of chromosome 6A (Wang, 2017), but it was inconsistent with the 6A QTL in this study. A QTL hotspot region for VI and GI (at physical position 9.2 Mb) was identified on chromosome 6B in a recombinant inbred line population derived from the cross of Chuan 355050/Shannong 483. It is tightly linked with markers *wpt8412* and *wmc487* and explains 7.41% and 9.98% of the phenotypic variances (Jiang et al., 2013), and was either not consistent with the 6B QTL in this study. Similarly, another QTL hotspot region for VI and GI was mapped on chromosome 6B and was linked with the markers *AX-110928656* and *AX-109883174* in the physical interval of 701.69-703.05 Mb (Shi et al., 2020), locating at a different physical position to the 6B QTL in this study. Consequently, the QTL hotspot regions for seed vigor related traits identified on chromosomes 6A and 6B are likely new loci.

## 5 Conclusion

In this study, 28 QTLs for seed vigor related traits were identified, accounting for 3.6%-23.7% of the phenotypic variances. Four new QTL clusters for seed vigor-related traits were mapped on chromosomes 4D, 5B, 6A, and 6B, respectively. The QTL on chromosome 4D was most likely to be *RHT-D1*. Meanwhile, we also developed a KASP marker for the QTL cluster *QVI.haust-4D.1/QSL.haust-4D.2/QRL.haust-4D*, and validated in an international panel of 135 wheat accessions. This study provided genes and molecular markers for improvement of seed vigor in wheat.

## Data availability statement

The original contributions presented in the study are included in the article/Supplementary Material. Further inquiries can be directed to the corresponding authors.

## Author contributions

CW designed the experiments. CG conducted phenotypic trait evaluations. ZZ conducted statistical analysis and wrote the paper. CW revised the manuscript. All authors contributed to the article and approved the submitted version.

## Funding

This work was financially supported by National Key Research and Development Plan for the “Thirteenth Five-Year Plan” (2018YFD0100904), Natural Science Foundation of Henan Province (162300410077), and International Cooperation Project of Henan Province (172102410052).

## Acknowledgments

We are grateful to Profs. Ravi Singh and Xianchun Xia for critical review of this manuscript.

## Conflict of interest

The authors declare that the research was conducted in the absence of any commercial or financial relationships that could be construed as a potential conflict of interest.

## Publisher’s note

All claims expressed in this article are solely those of the authors and do not necessarily represent those of their affiliated organizations, or those of the publisher, the editors and the reviewers. Any product that may be evaluated in this article, or claim that may be made by its manufacturer, is not guaranteed or endorsed by the publisher.
